# Aspirin Use among Adults with Cardiovascular Disease in the United States: Implications for an Intervention Approach

**DOI:** 10.3390/jcm8020264

**Published:** 2019-02-20

**Authors:** Benjamin E. Ansa, Zachary Hoffman, Nicollette Lewis, Cassandra Savoy, Angela Hickson, Rebecca Stone, Tara Johnson

**Affiliations:** 1 Institute of Public & Preventive Health, Augusta University, Augusta, GA 30912, USA; bstone@augusta.edu; 2 Master of Science in Experimental Psychology Program, Augusta University, Augusta, GA 30912, USA; zhoffma1@augusta.edu; 3 Medical College of Georgia, Augusta University, Augusta, GA 30912, USA; nilewis@augusta.edu; 4 Burke Medical Center of Waynesboro, Waynesboro, GA 30830, USA; swtcheeks_1966@yahoo.com; 5 Master of Public Health Program, Augusta University, Augusta, GA 30912, USA; ahickso3@augusta.edu; 6 Belvedere Elementary School, Aiken County Public School District, N. Augusta, SC 29841, USA; tjohnson3@acpsd.net

**Keywords:** aspirin, cardiovascular disease, angina, myocardial infarction, stroke, prevalence, United States, BRFSS

## Abstract

Cardiovascular disease (CVD) is a major underlying cause of death, with high economic burden in most countries, including the United States. Lifestyle modifications and the use of antiplatelet therapy, such as aspirin, can contribute significantly to secondary prevention of CVD in adults. This study examined the prevalence and associated factors of aspirin use for the secondary prevention of angina pectoris, myocardial infarction (MI), and cerebrovascular disease (stroke) in a sample of American adults. The 2015 Behavioral Risk Factor Surveillance System (BRFSS) dataset was analyzed for this cross-sectional study. Almost 16% of the study population (*N* = 441,456) had angina, MI, or stroke. Weighted percentages of respondents with angina, MI, and stroke were 4%, 4.3%, and 3%, respectively. Overall, weighted prevalence of daily (or every other day) aspirin use was about 65%, 71%, and 57% among respondents with angina, MI, and stroke, respectively. Factors that were significantly associated with aspirin use included male sex, more than high school education, high blood pressure, diabetes, and less than excellent general health. There were existing differences among individuals with CVD based on diagnosis, demographic and socioeconomic status in the use of aspirin for secondary prevention. Resources for promoting aspirin use should be directed toward groups with lower utilization.

## 1. Introduction

Cardiovascular disease (CVD) is a term for a number of disorders affecting the heart and blood vessels, which includes coronary heart disease (CHD), myocardial infarction, cerebrovascular disease, peripheral arterial disease, rheumatic and congenital heart diseases, and venous thromboembolism [1,2,3]. CVD has been listed as the major underlying cause of death in most countries. Globally, more than 17.3 million deaths due to CVD were estimated in 2013, accounting for 31% of all global mortality. This number is expected to grow to more than 23.6 million deaths by 2030 [1,2]. More than 92 million American adults have a diagnosis of CVD, with nearly 801,000 mortality cases, representing 1 out of every 3 deaths [4]. The direct and indirect costs of CVD and stroke in America are estimated to total more than $316 billion; this includes both health expenditures and lost productivity [4]. In 2010, the estimated global cost of CVD was $863 billion, and it is estimated to rise to $1044 billion by 2030 [4].

The risk of CVD can be reduced through lifestyle modifications, such as engaging in recommended physical activity; consuming healthy diets low in sugars and saturated fat and high in vegetables, fruits, and whole grains; avoiding smoking; reducing alcohol intake; and maintaining a healthy weight [5,6]. Antiplatelet therapy with agents such as aspirin is a major contributor to secondary prevention of CVD [2]. The cardioprotective benefits of aspirin result from its ability to cause the irreversible acetylation of cyclooxygenase (COX-1 enzyme) in platelets, thereby inhibiting the formation of prostaglandins and thromboxane A2, two potent promoters of platelet aggregation and vasoconstriction [7,8,9,10]. These findings are achieved by daily doses of 75 mg (and higher) or perhaps doses as low as 30 mg. Low-dose aspirin regimens (≥30 mg/day) can effectively suppress platelet aggregation without affecting important endothelial cell functions [8,11].

The U.S. Preventive Services Task Force (USPSTF) and other health-related agencies recommend aspirin use for the primary and secondary prevention of CVD in adults based on recognized risks, such as older age, male sex, race/ethnicity, abnormal lipid levels, high blood pressure, diabetes, and smoking status [12,13,14,15,16]. Although the role of aspirin for primary CVD prevention remains controversial [8], aspirin use for secondary prevention of CVD causes a significant reduction in nonfatal reinfarction, stroke, 5-week vascular mortality, and all-cause mortality [17]. Low-dose aspirin reduces the risk of vascular events (myocardial infarction (MI), stroke, and vascular death) in patients who have experienced an MI or a stroke or who are at high risk of vascular disease by Framingham risk score [18].

Several studies have examined the prevalence of aspirin use for the prevention of CVD as a group of disorders, but very few have examined its use for the prevention of specific disorders grouped under CVD. This study has three aims: (1) to examine the prevalence of CVD (angina pectoris, myocardial infarction, and cerebrovascular disease (stroke)) in a sample of American adults; (2) to examine the use of aspirin for the secondary prevention of angina pectoris, myocardial infarction, and cerebrovascular disease (stroke); and (3) to identify the factors associated with aspirin use among adults with CVD.

## 2. Materials and Methods

### 2.1. Study Design

The 2015 Behavioral Risk Factor Surveillance System (BRFSS) dataset was analyzed for this cross-sectional study.

### 2.2. Data Source, Study Participants, and Sampling

The BRFSS is a nationally representative cross-sectional survey that collects data on U.S. residents in all 50 states, the District of Columbia, and three U.S. territories regarding their health-related risk behaviors, chronic health conditions, and use of preventive services [19]. The BRFSS is a state-based, random-digit-dialed telephone survey of the noninstitutionalized U.S. civilian population aged 18 years or older [19,20]. Surveys are conducted through phone interviews (landline and cellphone), and more than 400,000 adult interviews are conducted each year, making it the largest continuously conducted health survey system in the world and a useful tool for addressing and developing health promotion activities [19,21]. Although conducted in different time periods, the surveys used identical methods for recruitment. Response rates for BRFSS were calculated using standards set by the American Association of Public Opinion Research (AAPOR) Response Rate Formula 4 [22]. The median survey response rate (%) for all states and Washington, DC, was 47.2 in 2015 and ranged from 33.9 to 61.1 [23].

### 2.3. Measures

Respondents were categorized under sociodemographic variables of sex (male or female); age in years (18–34, 35–44, 45–64, or ≥65); race (non-Hispanic (NH) white, NH black, Hispanic, other, or unknown); education (<high school, high school/General Educational Development (GED), >high school); annual income in United States dollar (USD (<$25,000, $25,000–$50,000 and >$50,000)); marital status (single relationship (divorced, widowed, separated, never married) and couple relationship (married or a member of an unmarried couple)); and healthcare coverage (yes/no). Smoking status was categorized as 1 every day, 2 some days, and not at all. Clinical characteristics included general health (poor/fair, good/very good, excellent); high blood pressure (yes/no); diabetes (yes/no); cholesterol (yes/no); and body mass index (BMI) (underweight, normal weight, overweight, obese). The outcome variables were participants’ response to the following questions: “Do you take aspirin daily or every other day?” (yes/no); “Have you ever been told by a doctor, nurse, or other health professional that you had a heart attack, also called a myocardial infarction?”(yes/no); “Have you ever been told by a doctor, nurse, or other health professional that you had angina or coronary heart disease?” (yes/no); and “Have you ever been told by a doctor, nurse, or other health professional that you had a stroke?” (yes/no).

### 2.4. Statistical Analyses

Descriptive statistics of sociodemographic and clinical variables related to CVD status and aspirin use were generated for 2015 using frequencies and proportions. Data were weighted using the iterative proportional fitting weighting method (i.e., raking) to adjust for noncoverage, nonresponse, and for differences between sample and population characteristics [24]. Raking is a method for adjusting the sampling weights of the sample data based on known population characteristics. Weighting is used to adjust the results of a study to bring them more in line with what is known about a population [25]. Weighted percentages of respondents who reported having CVD and use aspirin were calculated for each variable category. A weight is computed for every respondent in a sample, and it is computed by dividing the correct proportion by the observed proportion [25]. Logistic regression analyses were conducted to examine the association between sociodemographic variables and aspirin use among CVD respondents. Data were adjusted for sociodemographic and clinical status. Odds ratios and related 95% confidence intervals were derived from regression analysis. Chi-square test was used to obtain *p*-values. The significance level was set at *p* < 0.05, and all tests were two-sided. Unweighted counts, weighted percentages, and logistic regression analyses were performed using the IBM SPSS version 25 (IBM Corp., Armonk, NY, USA) [26].

### 2.5. Ethical Considerations

BRFSS datasets are publicly accessible and do not contain personally identifiable information. The Centers for Disease Control and Prevention (CDC) ensures that the process of data collection and release are governed by appropriate rules, regulations, and legislative authorizations [27].

## 3. Results

### 3.1. Sociodemographic and Clinical Characteristics of the Study Population

In the 2015 BRFSS database, a total of 441,456 adults responded to the question “Have you ever been told by a doctor, nurse, or other health professional that you had a heart attack (MI), angina, or stroke?” The respondents were ≥18 years old, predominantly female (57.7%), NH white (76.1%), between 45 and 64 years old (38.6%), with greater than high school education (63.9%), in a couple relationship (55.7%), with an annual income >$50,000 (39.3%), and had healthcare coverage (92.3%) (Table 1). A majority reported good/very good general health (63.9%), no high blood pressure (59.3%), no diabetes (84.3%), and no high blood cholesterol level (49.6%). For BMI and smoking status, there were 405,058 and 184,193 respondents, respectively. A majority of respondents were overweight (36.3%) and did not smoke at all (66.4%).

About 15.6% (*N* = 69,031) of respondents had CVD. More men reported having angina (53.3%) and MI (56.9%), while more women reported having stroke (58.3%). The majority of respondents (>60%) that reported a diagnosis of angina, MI, or stroke were 65 years and older. The results of the descriptive analyses of sociodemographic and clinical categories by CVD status were statistically significant.

### 3.2. Weighted Percentages of Respondents with Cardiovascular Disease

The prevalence of angina and MI was higher than that of stroke. The prevalence of overall angina, MI, and stroke were 4.0%, 4.3%, and 3.0%, respectively (Table 2). Men had higher prevalence (between 4% and 6%) of angina and MI, while women had higher prevalence of stroke (3.1%). CVD prevalence increased with age, and respondents 65 years and older had the highest prevalence. Angina (4.7%) and MI (4.8%) were reported more among NH white, while NH black reported more cases of stroke (4.1%). Prevalence of CVD decreased with increasing levels of education and income. Also, more cases of CVD were reported among individuals with high blood pressure, high blood cholesterol, and diabetes. Obese and overweight respondents and those reporting poor/fair general health had higher proportions of CVD.

### 3.3. Weighted Percentages of Respondents with Cardiovascular Disease that Take Aspirin Daily or Every Other Day

Overall, aspirin use was higher among respondents with MI (71.1%) than among those with angina (64.8%) and stroke (57.3%) (Table 3). Aspirin use was also higher among respondents that were males, ≥65 years, with more than high school education, and in a couple relationship. A majority of aspirin users with angina were NH white (75.3%), and a majority of aspirin users with stroke were NH black (69.8%). Also, aspirin use was highest among those with angina and MI earning annual income >$50,000 USD (>82%), while aspirin use was highest among those with stroke earning annual income of $25,000–$50,000 USD (64.8%). Most respondents with the three forms of CVD that were aspirin users either had high blood pressure (>63%) or diabetes (>71%). The least reported use of aspirin was among individuals with angina that were underweight (31.7%), with MI and stroke that had normal weight, and with the three forms of CVD that had excellent general health.

### 3.4. Multivariate Logistic Regression Analysis of Factors Associated with Aspirin Use

Angina, MI, and stroke were grouped together as one class of CVD, and the factors associated with aspirin use were determined from multivariate logistic regression analysis (Table 4). Adjusted odds ratio (aOR), 95% confidence interval, and *p*-values were calculated. Statistically significant determinants of aspirin use among respondents with CVD included sex, education, high blood pressure, diabetes, and general health. Females and respondents with less than high school education were less likely to use aspirin compared with males and respondents with more than high school education (aOR = 0.62; *p* < 0.0001, and aOR = 0.62; *p* = 0.03, respectively). Also, respondents with CVD who have high blood pressure and diabetes were more likely to use aspirin compared with those without the disorders (aOR = 1.46; *p* < 0.01, and aOR = 1.54; *p* < 0.01, respectively). Those that reported poor/fair general health and good/very good general health were 6.07 times (*p* < 0.001) and 2.77 times (*p* < 0.01) more likely to use aspirin compared with those with excellent general health.

## 4. Discussion

The 2015 BRFSS dataset was analyzed to examine the use of aspirin for the secondary prevention of CVD and the associated factors among adults with CVD in the United States. The prevalence of three forms of CVD (angina pectoris, myocardial infarction, and cerebrovascular disease (stroke)) in a sample of American adults was also determined. Almost 16% of the study population had angina, MI, or stroke. Weighted percentages of respondents with angina, MI, and stroke were 4%, 4.3%, and 3%, respectively. Overall, weighted prevalence of daily (or every other day) aspirin use was about 65%, 71%, and 57% among respondents with angina, MI, and stroke, respectively. Aspirin use among respondents with CVD was associated with male sex, more than high school education, high blood pressure, diabetes, and less than excellent general health.

The results of the current study are similar to those reported by Luepker et al. [28]. Men were more likely to use aspirin than women at any age, and aspirin use was highest in those aged 65 to 74 years. Jing Fang et al. [29] examined the 2013 BRFSS data for the use of aspirin for the secondary prevention of atherosclerotic CVD among adults in 20 states and the District of Columbia in the United States and revealed that 12.5% of respondents had coronary heart disease, stroke, or both, and about 71% used aspirin. Almost 94%, 80%, and 76% took aspirin to prevent heart attack, stroke, or both, respectively. Also, aspirin use was more likely among males, NH white, and 65 years and older. A previous study by Jing Fang et al. [30] using the 2012 National Health Interview Survey also showed that men, 65 years and older, and NH whites were significantly more likely to take low-dose aspirin for secondary prevention and that hypertension, diabetes, high blood cholesterol, and current smoking were related to low-dose aspirin use. In contrast to the current study, race and smoking status were not significantly associated with aspirin use. Results from Mendy et al. [20] showed that, in Mississippi, United States, 18.9% of men and 17.8% of women reported having CVD, and among those with CVD, 85.9% of men and 85.1% of women reported taking aspirin. Greater than high school education and diabetes were significantly associated with aspirin use for secondary prevention of CVD among women, while only the white race was the associated factor in men. Analysis of the 2000–2002 Medical Expenditure Panel Surveys by Opotowsky et al. [31] showed that women (62.4% vs. 75.6% men) and those without hypertension (66.1% vs. 72.1% with hypertension) were less likely to use aspirin regularly, and in contrast to the current study, diabetes was not significantly associated with aspirin use. Stuntz et al. [32] presented results using data from the 2012–2015 National Health Interview Survey and revealed coronary heart disease as the most common CVD (7.1%), followed by myocardial infarction (5.0%), stroke (4.1%), and angina pectoris (3.0%). Prevalence of aspirin use was highest among males, 65 years and older, NH blacks, living in the Midwest region, with household annual income greater than $35,000, with health insurance, obese, and with less than high school education. Body mass index was not associated with aspirin use in the current study.

The current report reveals existing differences based on diagnosis in the use of aspirin for secondary prevention among individuals with CVD. More men reported using aspirin than women. One possible explanation for the gender differences is that women are more likely to report contraindications to aspirin use than men [31,32,33,34]. Gender differences in aspirin use could also be explained by uncertainty and related biases among treating physicians, since providers may generalize the unclear efficacy of aspirin in primary prevention for women at low risk of vascular events to women with known CVD [33,34]. The prevalence of aspirin use was also highest among those with MI and least for those with stroke. Hemorrhagic stroke may have been a contraindication for aspirin use, hence the lower prevalence among respondents with stroke. Compared to individuals with higher educational attainment, those with less than high school education had a much higher proportion of CVD but lesser proportion of aspirin utilization. The reason for this observation is worth further investigation. Could this be taken as evidence for the cardioprotective effect of aspirin? Or is lower educational (and social) status a barrier to aspirin access?

The role of aspirin for individuals without CVD is more controversial [35], and the risk of bleeding complications from aspirin ingestion has called into question the level of baseline CVD risk for which use of aspirin in primary prevention is clinically acceptable [36]. However, more than 84% of the current study population did not report having any form of CVD and less than 32% took aspirin for primary prevention (results not shown). Mendy et al. [20] also observed that, among those without CVD, 39.1% of men and 45.9% of women reported taking aspirin. These results probably reflect the controversy surrounding aspirin use for primary prevention of CVD.

There are limitations to the interpretation of data from the current study. Only three forms of CVD were examined for prevalence of aspirin use. The BRFSS data did not include other forms of CVD, such as peripheral arterial disease and deep venous thrombosis, and therefore these results may not be generalized to include these cases. Also, the questions for identifying persons with CVD (e.g., “Have you ever been told by a doctor, nurse, or other health professional that you had a heart attack, also called a myocardial infarction?”) did not include individuals with asymptomatic CVD, without previous thrombotic episodes. Errors in the true estimates of aspirin use among women and respondents with stroke may have occurred as a result of contraindications to aspirin use that were not factored into the analyses. It was not possible to differentiate between thromboembolic and hemorrhagic stroke in the BRFSS data that was used for this study. This may partially account for the lower reported prevalence of aspirin use for secondary prevention of stroke compared with heart attack. Limiting the analysis to those without a contraindication to aspirin use may narrow the absolute gender gap [31]. Recall bias from self-reporting may have caused overestimation, underestimation, or misclassification of the results presented. Despite these limitations, data from the BRFSS are reliable and generally valid because the content of the survey questions, questionnaire design, data collection, procedures, interviewing techniques, and data processing have been developed to improve data quality [37]. Data from this study can be helpful to clinicians and public health practitioners in directing resources for promoting aspirin use toward groups with lower prevalence.

## 5. Conclusions

Regular use of aspirin for secondary prevention of CVD has been proven to cause a significant reduction in morbidity and mortality attributed to CVD. However, not all groups of individuals are leveraging the benefits from the recommended use of aspirin and may consequently be at higher risks for cardiovascular events and premature death. Further research is needed to better understand the underlying causes of lower rates of aspirin use among identified individuals with CVD. Strategies to increase aspirin use may include making policies that promote utilization at all levels of primary and public health care, educating patients and caregivers about the benefits of aspirin, and implementing interventions that target the underserved.

## Figures and Tables

**Table 1:** Sociodemographic and clinical characteristics of the study population by CVD Status: BRFSS 2015 data.

Variables	Total *N* = 441,456 (%)	No CVD *N* = 372,425 (%)	Cardiovascular Disease (CVD) *N* =69,031 (%)		
			Angina *N* = 25,290 (%)	Myocardial Infarction *N* = 25,472 (%)	Stroke *N* = 18,269 (%)
Sex					
Male	186,938 (42.3)	151,350 (40.6)	13,490 (53.3)	14,484 (56.9)	7,614 (41.7) *
Female	254,518 (57.7)	221,075 (59.4)	11,800 (46.7)	10,988 (43.1)	10,655 (58.3) *
Age (years)					
18–34	66,915 (15.2)	65,988 (17.7)	274 (1.1)	313 (1.2)	340 (1.9)
35–44	50,819 (11.5)	49,033 (13.2)	487 (1.9)	646 (2.5)	653 (3.6)
45–64	170,513 (38.6)	149,048 (40.0)	7618 (30.1)	7939 (31.2)	5908 (32.3)
65+	153,209 (34.7)	108,356 (29.1)	16,911 (66.9)	16,574 (65.1)	11,368 (62.2)
Race					
NH white	336,066 (76.1)	281,672 (75.6)	20,430 (80.8)	20,232 (79.4)	13,732 (75.2)
NH black	34,346 (7.8)	28,616 (7.7)	1758 (7.0)	1872 (7.4)	2100 (11.5)
Hispanic	35,795 (8.1)	32,194 (8.6)	1354 (5.4)	1384 (5.4)	863 (4.7)
Other	27,812 (6.3)	23,850 (6.4)	1301 (5.0)	1474 (5.8)	1187 (6.5)
Unknown	7437 (1.7)	6093 (1.6)	447 (1.8)	510 (2.0)	387 (2.1)
Education					
<High school	34,259 (7.8)	24,994 (6.7)	2930 (11.6)	3622 (14.2)	2713 (14.9)
High school	123,227 (27.9)	100,225 (26.9)	8066 (31.9)	8790 (34.5)	6146 (33.6)
>High school	282,159 (63.9)	245,666 (66.0)	14,204 (56.1)	12,951 (50.8)	9338 (51.1)
Don’t know/Refused	1811 (0.4)	1540 (0.4)	90 (0.4)	109 (0.4)	72 (0.4)
Income					
<$25K	97,222 (22.0)	72,871 (19.6)	8190 (32.4)	9037 (35.5)	7124 (39.0)
25K–50K	91,287 (20.7)	75,773 (20.3)	5817 (23.0)	5815 (22.8)	3882 (21.2)
>50K	173,442 (39.3)	157,446 (42.3)	6755 (26.7)	5771 (22.7)	3470 (19.0)
Don’t know/Refused	79,505 (18.0)	66,335 (17.8)	4528 (17.9)	4849 (19.0)	3793 (20.8)
Marital status					
Single relationship	192,523 (43.6)	156,520 (42.1)	12,391 (49.0)	13,167 (51.7)	10,445 (57.2)
Couple relationship	245,837 (55.7)	213,125 (57.2)	12,797 (50.6)	12,194 (47.9)	7721 (42.3)
Don’t know/Refused	3096 (0.7)	2780 (0.7)	102 (0.4)	111 (0.4)	103 (0.6)
Health coverage					
Yes	407,556 (92.3)	341,549 (91.7)	24,377 (96.4)	24,275 (95.3)	17,355 (95.0)
No	32,060 (7.3)	29,233 (7.8)	861 (3.4)	1116 (4.4)	850 (4.7)
Don’t Know/Refused	1840 (0.4)	1643 (0.5)	52 (0.2)	81 (0.3)	64 (0.3)
General health					
Poor/Fair	82,137 (18.6)	47,898 (12.9)	12,612 (49.9)	12,527 (49.2)	9100 (49.8)
Good/Very good	282,040 (63.9)	250,317 (67.2)	11,708 (46.3)	11,756 (46.1)	8259 (45.2)
Excellent	76,032 (17.2)	73,327 (19.7)	867 (3.4)	1044 (4.1)	794 (4.3)
Don’t know/Refused	1247 (0.3)	883 (0.2)	103 (0.4)	145 (0.6)	116 (0.7)
High blood pressure					
Yes	178,188 (40.4)	126,451 (34.0)	19,517 (77.2)	18,818 (73.9)	13,402 (73.4)
No	261,829 (59.3)	245,402 (65.8)	5490 (21.7)	6319 (24.8)	4618 (25.3)
Others/Don’t know	1439 (0.3)	572 (0.2)	283 (1.1)	335 (1.3)	249 (1.3)
Diabetes					
Yes	57,256 (13.0)	34,630 (9.3)	8541 (33.8)	8526 (33.5)	5559 (30.4)
No	372,104 (84.3)	328,019 (88.1)	15,901 (62.9)	16,113 (63.2)	12,071 (66.1)
Others/Don’t know	12,096 (2.7)	9776 (2.6)	848 (3.3)	833 (3.3)	639 (3.5)
Cholesterol	*N* = 441,456	*N* = 375,195	*N* = 24,623	*N* = 24,431	*N* = 17,207
Yes	159,970 (36.2)	114,752 (30.6)	17,754 (72.1)	16,637 (68.1)	10,827 (62.9)
No	218,771 (49.6)	198,513 (52.9)	6628 (26.9)	7493 (30.7)	6137 (35.7)
Don’t know/Refused	62,715 (14.2)	61,930 (16.5)	241 (1.0)	301 (1.2)	243 (1.4)
Smoking status	N=184,193	*N* = 143,474	*N* = 14,770	*N* = 15,783	*N* = 10,166
1 every day	43,583 (23.7)	35,285 (24.6)	2479 (16.8)	3403 (21.6)	2416 (23.8)
2 some days	17,998 (9.8)	14,599 (10.2)	1,058 (7.2)	1,301 (8.2)	1040 (10.2)
Not at all	122,277 (66.4)	93,348 (65.1)	11,197 (75.8)	11,035 (69.9)	6697 (65.9)
Don’t know/Refused	335 (0.2)	242 (0.1)	36 (0.2)	44 (0.3)	13 (0.1)
BMI	*N* = 405,058	*N* = 339,863	*N* = 23,959	*N* = 24,103	*N* = 17,133
Underweight	6721 (1.7)	5570 (1.6)	335 (1.4)	419 (1.7)	397 (2.3)
Normal weight	131,409 (32.4)	115,264 (33.9)	5534 (23.1)	5850 (24.3)	4761 (27.8)
Overweight	147,004 (36.3)	122,928 (36.2)	8967 (37.4)	9020 (37.4)	6089 (35.5)
Obese	119,924 (29.6)	96,101 (28.3)	9123 (38.1)	8814 (36.6)	5886 (34.4)

BRFSS: Behavioral Risk Factor Surveillance System, NH: non-Hispanic. * All *p*-values < 0.0001, except for sex vs. stroke (*p*-value = 0.007).

**Table 2:** Weighted percentages of respondents with cardiovascular disease: BRFSS 2015.

Variables	Cardiovascular Disease			*p*-Value
	Angina Weighted *N* (%)	Myocardial Infarction Weighted *N* (%)	Stroke Weighted *N* (%)	
Overall	10,154,549 (4.0)	10,691,710 (4.3)	7,609,840 (3.0)	
Sex				<0.0001
Male	5,883,465 (4.8)	6,741,115 (5.5)	3,605,072 (2.9)	
Female	4,271,084 (3.3)	3,950,595 (3.1)	4,004,768 (3.1)	
Age				<0.0001
18–34	290,818 (0.4)	340,991 (0.5)	360,840 (0.5)	
35–44	433,773 (1.1)	590,022 (1.4)	526,960 (1.3)	
45–64	3,777,164 (4.4)	4,134,347 (4.9)	2,971,101 (3.5)	
65+	5,652,794 (11.5)	5,626,350 (11.4)	3,750,939 (7.6)	
Race				<0.0001
NH white	7,342,743 (4.7)	7,568,147 (4.8)	5,049,650 (3.2)	
NH black	1,008,762 (3.5)	1,117,769 (3.9)	1,190,126 (4.1)	
Hispanic	1,039,482 (2.5)	1,160,996 (2.8)	732,644 (1.8)	
Other	604,787 (3.1)	636,153 (3.2)	480,468 (2.4)	
Unknown	158,775 (3.8)	208,644 (4.9)	156,951 (3.7)	
Education				<0.0001
<High school	2,078,745 (5.8)	2,626,453 (7.3)	1,873,946 (5.2)	
High school	3,133,312 (4.4)	3,481,348 (4.9)	2,429,170 (3.4)	
>High school	4,917,798 (3.4)	4,543,369 (3.2)	3,279,577 (2.3)	
Income				<0.0001
<$25K	3,346,520 (5.6)	3,945,885 (6.6)	3,055,341 (5.1)	
$25K–$50K	2,375,159 (4.7)	2,354,763 (4.7)	1,599,694 (3.2)	
>$50K	2,709,241 (2.8)	2,405,748 (2.5)	1,419,673 (1.5)	
Marital status				<0.0001
Single relationship	4,479,142 (4.0)	5,118,891 (4.6)	3,984,256 (3.6)	
Couple relationship	5,631,915 (4.1)	5,525,284 (4.0)	3,582,067 (2.6)	
High blood pressure				<0.0001
Yes	7,718,064 (9.6)	7,745,099 (9.7)	5,478,837 (6.8)	
No	2,344,322 (1.4)	2,812,256 (1.7)	2,037,462 (1.2)	
Smoking				<0.0001
1 every day	1,112,518 (4.1)	1,640,283 (6.0)	1,188,411 (4.3)	
2 some days	532,100 (4.3)	640,950 (5.1)	490,845 (3.9)	
Not at all	4,325,414 (7.4)	4,373,889 (7.5)	2,606,681 (4.5)	
Cholesterol				<0.0001
Yes	6,983,293 (9.7)	6,807,803 (9.5)	4,343,065 (6.0)	
No	2,707,512 (2.2)	3,138,353 (2.5)	2,625,566 (2.1)	
Diabetes				<0.0001
Yes	3,516,016 (13.3)	3,656,625 (13.9)	2,286,880 (8.7)	
No	6,246,247 (2.9)	6,657,234 (3.1)	5,027,487 (2.3)	
BMI				<0.0001
Underweight	139,326 (3.4)	166,135 (4.0)	156,285 (3.8)	
Normal weight	2,145,210 (2.8)	2,343,929 (3.0)	1,952,532 (2.5)	
Overweight	3,519,681 (4.3)	3,741,929 (4.6)	2,509,450 (3.1)	
Obese	3,791,843 (5.7)	3,853,416 (5.8)	2,469,480 (3.7)	
General health				<0.0001
Poor/Fair	5,220,703 (11.8)	5,483,745 (12.4)	3,921,665 (8.8)	
Good/Very good	4,505,342 (2.8)	4,668,229 (2.9)	3,261,927 (2.0)	
Excellent	387,613 (0.8)	481,190 (1.0)	381,046 (0.8)	

**Table 3:** Weighted percentages of respondents with cardiovascular disease that take aspirin daily or every other day: BRFSS 2015.

Variables	Aspirin Use			*p*-Value
	Angina *N* (%)	Myocardial Infarction *N* (%)	Stroke *N* (%)	
Overall	406,221 (64.8)	400,916 (71.1)	236,207 (57.3)	
Sex				<0.0001
Male	239,240 (71.3)	258,053 (72.2)	124,831 (60.1)	
Female	166,981 (57.4)	142,864 (72.0)	111,376 (54.4)	
Age				<0.0001
18–34	6064 (26.7)	3114 (18.4)	2350 (23.8)	
35–44	18,835 (45.3)	9598 (50.3)	18,111 (38.6)	
45–64	158,054 (62.8)	177,984 (72.9)	96,543 (56.8)	
65+	223,268 (71.9)	210,220 (76.2)	119,203 (64.2)	
Race				<0.0001
NH white	253,540 (75.3)	259,428 (79.1)	147,733 (58.5)	
NH black	40,408 (68.7)	52,729 (79.8)	46,027 (69.8)	
Hispanic	12,801 (56.0)	69,728 (51.7)	32,928 (49.4)	
Other	94,962 (47.6)	14,003 (68.9)	7556 (32.9)	
Unknown	4510 (54.0)	5028 (74.0)	1964 (42.7)	
Education				<0.0001
<High school	93,621 (60.1)	107,552 (67.0)	66,381 (61.3)	
High school	102,544 (59.8)	112,487 (69.4)	75,024 (55.4)	
>High school	208,325 (70.1)	180,368 (77.7)	94,516 (56.3)	
Income				<0.0001
<$25K	169,511 (57.6)	178,288 (70.2)	102,862 (57.8)	
$25K–$50K	84,218 (76.5)	85,257 (74.8)	59,350 (64.8)	
>$50K	87,213 (82.4)	75,320 (82.7)	36,810 (51.6)	
Marital status				<0.0001
Single relationship	158,617 (59.4)	173,558 (70.2)	99,261 (54.0)	
Couple relationship	245,095 (68.7)	225,195 (73.7)	136,440 (59.9)	
High blood pressure				<0.0001
Yes	344,584 (66.4)	327,861 (73.9)	192,508 (63.0)	
No	60,857 (58.2)	71,196 (65.2)	41,968 (41.9)	
Smoking				<0.0001
1 every day	42,814 (64.5)	54,670 (71.7)	30,290 (55.1)	
2 some days	14,002 (49.2)	17,855 (61.9)	16,538 (50.6)	
Not at all	178,082 (72.1)	195,107 (79.5)	90,360 (63.7)	
Cholesterol				<0.0001
Yes	281,221 (69.3)	262,961 (75.1)	139,140 (58.3)	
No	112,368 (60.5)	112,913 (70.4)	80,942 (59.4)	
Diabetes				<0.0001
Yes	178,506 (75.4)	172,219 (75.7)	88,971 (71.6)	
No	210,691 (58.3)	212,351 (70.0)	136,382 (50.8)	
BMI				<0.0001
Underweight	3525 (31.7)	14,361 (76.8)	2617 (61.9)	
Normal weight	83,675 (66.2)	75,671 (67.3)	51,519 (46.8)	
Overweight	145,449 (64.6)	144,373 (74.5)	76,820 (63.4)	
Obese	163,510 (67.5)	153,999 (71.9)	97,121 (61.3)	
General health				<0.0001
Poor/Fair	244,754 (62.9)	124,095 (76.0)	122,761 (60.6)	
Good/Very good	146,516 (68.7)	34,252 (68.1)	97,299 (56.3)	
Excellent	131,82 (60.7)	15,937 (56.5)	14,720 (41.7)	

**Table 4:** Multivariate logistic regression analysis of factors associated with aspirin use among respondents based on CVD status: BRFSS 2015 data.

Variables	Odds Ratio	95% Confidence Interval	*p*-Value
Sex			
Male	Ref.		
Female	0.62	0.49–0.79	<0.0001
Age (years)			
18–34	Ref.		
35–44	1.13	0.19–6.78	0.89
45–64	0.84	0.17–4.18	0.83
65+	1.24	0.24–6.17	0.79
Race/Ethnicity			
NH white	Ref.		
NH black	0.99	0.62–1.59	0.98
Hispanic	0.77	0.41–1.44	0.42
Other	0.90	0.30–2.65	0.85
Education			
>High school	Ref.		
High school	0.68	0.45–1.03	0.07
<High school	0.62	0.41–0.94	0.03
Income			
>$50K	Ref.		
$25K–$50K	0.91	0.67–1.24	0.55
<$25K	0.73	0.52–1.03	0.07
Marital status			
Single relationship	0.83	0.64–1.08	0.16
Couple relationship	Ref.		
Insurance			
Yes	Ref.		
No	0.69	0.35–1.37	0.29
High blood pressure			
No	Ref.		
Yes	1.46	1.11–1.93	<0.01
Smoking			
Not at all	Ref.		
1 every day	0.82	0.60–1.12	0.21
2 some days	1.03	0.63–1.66	0.92
Cholesterol			
No	Ref.		
Yes	1.17	0.91–1.51	0.22
Diabetes			
No	Ref.		
Yes	1.54	1.19–1.99	<0.01
BMI			
Underweight	Ref.		
Normal weight	0.45	0.15–1.34	0.15
Overweight	0.44	0.15–1.30	0.14
Obese	0.37	0.12–1.10	0.07
General health			
Excellent	Ref.		
Good/Very good	2.77	1.56–4.99	<0.01
Poor/Fair	6.07	3.29–11.18	<0.001
